# Screening for a false unipolarity in patients treated for a major depressive disorder

**DOI:** 10.1192/j.eurpsy.2021.533

**Published:** 2021-08-13

**Authors:** N. Regaieg, I. Baati, F. Ben Amor, M. Kallel, S. Hentati, J. Masmoudi

**Affiliations:** 1 Psychiatry “a” Department, Hedi Chaker UHC, Sfax, Tunisia; 2 Psychiatry “a” Department, Hedi Chaker University Hospital, Sfax, Tunisia; 3 Psychiatrie “a” Department, Hedi Chaker Hospital University -Sfax - Tunisia, sfax, Tunisia

**Keywords:** HCL-20, bipolar depression, bipolar II disorder, hypomania

## Abstract

**Introduction:**

The early diagnosis of bipolar II disorder remains difficult in clinical practice, hence the importance of psychometric tests.

**Objectives:**

To detect hypomania in patients followed for a major depressive disorder (MDD) and to determine factors which are correlated with it.

**Methods:**

This was a cross-sectional, descriptive and analytical study. It involved 40 psychiatric outpatients, who were followed for MDD (isolated or recurrent episode) at the Hedi Chaker University Hospital in Sfax (Tunisia), from January 26 to February 10, 2020. The study was conducted using a questionnaire and the Angst Hypomania Checklist-20 (HCL-20).

**Results:**

The sex ratio (M/F) was 0.66 with an average age of 54.8 years. MDD started at an average age of 41.45 years. According to HCL-20, half of our sample had hypomania. The presence of hypomania was correlated with young age (p = 0.022), academic failure (p = 0.038) and smoking (p = 0.003). In addition, there was a statistically significant relationship between the presence of hypomania and the characteristics of the disease: number of depressive episodes ≥ 2 (p = 0.013), psychotic features (p = 0.038), melancholic features (p=0,025) and premature discontinuation of treatment (p = 0.003).

**Conclusions:**

Our study confirmed that bipolar depression is still underdiagnosed and poorly treated. Questioning a patient about a history of hypomania would be a delicate task and would require the help of a scale, in particular the HCL -20.

